# Cue-based feeding and growth in preterm infants: a two-year follow-up study

**DOI:** 10.3389/fped.2026.1771033

**Published:** 2026-03-25

**Authors:** Iris Morag, Dina Kogan, Eden Weinberg, Daniella Darsa, Or Derin, Sagee Nissimov, Michal Yackobovitch-Gavan

**Affiliations:** 1 Shamir Medical Center, Affiliated with the Gray Faculty of Medicine, Tel Aviv University, Tel Aviv, Israel; 2 George S. Wise Faculty of Life Sciences, Tel Aviv University, Tel Aviv, Israel; 3 Department of Epidemiology & Preventive Medicine, The Open University of Israel, Raanana, Israel; 4 The Gray Faculty of Medical and Health Sciences, Tel Aviv University, Tel Aviv, Israel

**Keywords:** cue-based feeding, feeding difficulties, growth, NICU, parental involvement, preterm infants, responsive feeding

## Abstract

**Aim:**

To study the association between cue-based feeding (CBF) and growth trajectories and feeding characteristics up to two years of age.

**Methods:**

A retrospective cohort study of preterm infants born at 27⁰/₇−33⁶/₇ weeks’ gestation across two feeding epochs [traditional feeding (TF) vs. CBF]. Anthropometric indices were assessed at discharge and during routine follow-up. Feeding characteristics at two years were assessed using the Montreal Children's Hospital Feeding Scale (MCHFS).

**Results:**

The cohort included 115 infants (TF *n* = 58; CBF *n* = 57). CBF was associated with a shorter length of stay (mean difference 11.8 days, *p* < 0.001) and earlier discharge (35.5 vs. 36.9 weeks’ PMA, *p* < 0.001). Weight and head circumference *Z*-scores at discharge were comparable. Across follow-up, both groups demonstrated declines in weight- and length-for-age *Z*-scores, with consistently higher values in the TF group (weight: *p* = 0.004; length: *p* = 0.003). Total MCHFS scores and prevalence of feeding difficulties at two years were similar. However, coercive feeding behaviors were significantly more common in the TF group (*p* = 0.018 and *p* = 0.044).

**Conclusion:**

CBF was associated with earlier discharge and more adaptive parent-infant feeding interactions while supporting overall normal growth. Integrating responsive feeding with structured nutritional monitoring may optimize both somatic and relational outcomes in preterm infants.

## 1 Introduction

Feeding remains a major challenge in neonatal care. To meet metabolic demands, most stable growing preterm infants require fluid intake of 150–180 mL/kg/day and 115–140 kcal/kg/day to achieve appropriate nutrient intake (1, 2). The transition to oral feeding typically occurs at 32–34 weeks postmenstrual age (PMA) and requires balancing nutritional adequacy with physiologic and behavioral readiness (3–5).

Traditional, volume-driven feeding (TF) involves administering predefined milk volumes at fixed intervals. While TF ensures caloric sufficiency, it may override infant readiness cues and has been associated with stressful feeding experiences and feeding-related complications (6, 7). In contrast, cue-based feeding (CBF) is a responsive approach that individualizes oral feeding based on infant cues, allowing flexibility in feeding intervals and volumes (8, 9).

In our unit, CBF was implemented as a parental-guided responsive feeding model in which parents lead the interpretation of infant feeding cues. Infants are offered flexible feeding volumes and intervals, with partial supplementation via nasogastric tube when a predefined minimum volume was not achieved, in order to ensure nutritional adequacy. This model integrates principles of developmental care by actively positioning parents as primary partners in the feeding process. It is also consistent with international responsive feeding guidelines, which define feeding as a prompt, contingent, and developmentally attuned response to infant cues while avoiding intrusive or coercive strategies. Responsive feeding is recognized by the World Health Organization as a core component of early childhood nutrition and caregiving and is associated with emerging self-regulation, physiological stability, and positive caregiver-infant interaction patterns (10, 11). Within this framework, feeding is conceptualized not only as nutritional transfer but as a relational process that supports developmental organization.

CBF is conceptually aligned with developmental frameworks including the synactive theory (12, 13) introduced by Als and Pridham's guided participation model (14), both of which emphasize the infant's capacity for self-regulation within supportive caregiving relationships.

Implementation of CBF in neonatal intensive care units (NICU) has been associated with shorter time to full oral feeding, reduced nasogastric tube duration, and earlier discharge (15–18). Beyond initial hospitalization, feeding practices during infancy influence growth, emotional regulation, and emerging communication capacities (19). Early feeding difficulties in preterm infants, including those already evident at 30 months of age, have been associated with persistent eating problems and poorer growth trajectories in later childhood (20), and controlling feeding interactions has been linked with a higher risk of feeding disorders and developmental delays (21–26).

Nevertheless, evidence regarding the longer-term implications of CBF, particularly its effects on post-discharge growth trajectories and parent-infant feeding interactions, remains limited. Most existing studies have focused on short-term hospital outcomes (18, 27), leaving open the question of whether CBF confers benefits in feeding behavior and growth after discharge.

The aim of this study was therefore to compare growth indices and feeding characteristics up to two years of age among preterm infants who transitioned to oral feeding using CBF vs. those who transitioned using TF.

## 2 Materials and methods

### 2.1 Research ethics and consent

This single-center retrospective cohort study was conducted in a tertiary care NICU with approximately 8,000 births per year. The study was approved by the institutional review board's ethics and research committee (study reference no.0281-21-ASF). The committee ensured that the study adhered to all relevant guidelines and regulations. Given the retrospective design, informed consent for chart review was waived; however, parental completion of the follow-up questionnaire was considered to be consent to participate in the prospective component of the study.

### 2.2 Study design

CBF was introduced in March 2020. Accordingly, three epochs were defined: (a) TF: June 2018–February 2020; (b) transition period: March 2020–December 2020, (c) CBF: January–September 2021. Feeding approach was determined solely by the time period of hospitalization, as the NICU adopted cue-based feeding as a unit-wide protocol beginning January 2021, with no individual crossover between feeding strategies.

### 2.3 Setting

Preterm infants born at <35 weeks' gestation were routinely transferred to the NICU, with the majority being inborn and cared for by the same teams from birth to discharge.

### 2.4 Cue-based feeding

Under the CBF protocol, transition to oral feeding was guided by responsive practices involving both parents and caregivers, as previously described (9). Historically, preterm infants have been introduced to oral feeding between 32 and 34 weeks' PMA (28). Consistent with this range, initiation of oral feeding in our unit typically occurred between 32 and 34 weeks' once infants demonstrated neurological and physiological readiness, including: (a) hemodynamic and respiratory stability (non-invasive respiratory support was not a contraindication, and mechanically ventilated preterm infants in our unit are not routinely managed with continuous sedation) (b) presence of hunger cues such as rooting, mouthing, or hand-to-mouth activity; and (c) effective non-nutritive sucking on a pacifier.

No formal readiness assessment tool was applied. Instead, readiness and initiation of oral feeding were guided by ongoing clinical assessment within our parental-guided responsive feeding model, which emphasizes parental attunement to infant cues together with clinician evaluation of physiological stability and behavioral organization. This approach draws on responsive and cue-based feeding principles described by Lubbe and Shaker, which emphasize careful clinical observation, developmental support, and co-regulation within the caregiver-infant relationship (4–6). Readiness cues included an alert and organized behavioral state, rooting, mouthing, hand-to-mouth activity, effective non-nutritive sucking, and the ability to maintain cardiorespiratory stability during handling. Signs of stress or physiological instability (e.g., desaturation, bradycardia, or disengagement) prompted postponement or modification of feeding attempt. Human milk was prioritized, with direct breastfeeding encouraged as the primary feeding mode whenever feasible. Direct breastfeeding was considered from approximately 32 weeks' PMA based on clinical assessment of physiological stability and emerging feeding organization. Bottle feeding was introduced at 33 weeks' PMA for infants whose mothers did not intend exclusive breastfeeding and at 34 weeks for those planning exclusive breastfeeding, consistent with the “feeding imprinting” theory (29).

Feedings were offered every 2–4 h. During the transition to full oral feeding, daily intake ranged from 80 to 200 mL/kg/day. (approximately 10–25 mL/kg per meal, assuming 6–8 feeds per day). The lower range reflected a temporary minimum intake during a brief transitional phase and did not represent the intended long-term nutritional target. Feeding progression was individualized and guided by routine clinical assessment of total intake, weight trends, and overall physiological stability. Adjustments were made if intake did not improve within a few days. When oral intake did not reach the minimum expected threshold, nasogastric supplementation was provided up to ∼120 mL/kg/day (≈15 mL/kg/meal), rather than to the full calculated volume. Blood glucose was routinely monitored on the first two occasions when the feeding interval exceeded 3.5 h.

For directly breastfed infants, milk transfer was not measured quantitatively to avoid reliance on external parameters. Supplementation decisions were made once a day in collaboration between parents and the lactation consultant, based on maternal reports and clinical observations (latch quality, sucking pattern, and satiety cues).

Infants were weighed every 3–4 days per unit policy, with additional weighing on the day of feeding transition. If weight loss or hypoglycemia occurred, feeding intervals were shortened to 3–3.5 h or nasogastric supplements were increased beyond 120 mL/kg/day. Feeding methods were limited to direct breastfeeding, bottle feeding (introduced at 33–34 weeks), and nasogastric supplementation; cup feeding was not used.

### 2.5 Traditional feeding

During the pre-CBF epoch, feeding practices followed traditional, volume-driven protocols. Oral feeding was typically initiated at later postmenstrual ages than CBF (around 34 weeks) and consisted of predefined milk volumes administered every 3 h, with supplementation provided to meet prescribed daily totals. Bottle feeding was typically introduced earlier, and parental involvement in feeding decisions was minimal. Otherwise, medical management was consistent across epochs. These historical practices served as the comparative epoch for evaluation against the CBF protocol.

### 2.6 Study participants

Included were all preterm infants that were born between 27⁰/₇ and 33⁶/₇ weeks gestation and survived to discharge during study epochs 1 and 3. Excluded were infants with proven or suspected genetic syndromes, triplets, birthweight <3% according to Fenton growth charts (30), severe brain abnormalities (intra-ventricular hemorrhage grade >2 or periventricular leukomalacia) (31), proven necrotizing enterocolitis (31), or maternal conditions that were not compatible with the use of own mother breast milk. Also excluded were infants born during epoch 2 as it was considered an implementation period. Infants whose parents did not respond to calls or refused to participate in post discharge data collection were also excluded.

### 2.7 Data collection

Medical records were assessed for maternal and infant clinical and demographic characteristics: maternal age, marital status, previous deliveries, chronic medical conditions and medications, smoking, perinatal conditions, delivery mode, gestational age, sex at birth, Apgar scores, need for mechanical ventilation, duration of oxygen treatment, weight at birth and at discharge/36 weeks (whichever was first) and head circumference. The second step included post discharge data collection which involved contacting families via phone calls by one of the researchers (D.K) who was not familiar with the child's NICU medical course. We limited the contact attempts to five trials at different time points. Data were documented on a special collection form.

### 2.8 Outcome measures

Growth characteristics: Well-child health records in the country include regular anthropometric measurements for every child from birth to age six at national maternal child health clinics. Participating parents were requested to submit a copy of their child's anthropometric measurements. Weight, length, head circumference and calculated body mass index (BMI) at 12 (9–13), 18 (15–20) and 24 (24–32) month corrected age were analyzed. At birth, only weight and head circumference were measured, while head circumference was not routinely measured at >18 months visits. *Z*-scores for all growth indices were calculated using WHO charts (32), according to sex and corrected age. All infants born prior to 36 weeks' gestation are automatically referred for nutritional follow-up. This structured follow-up system is standardized nationwide and was applied consistently across both epochs.

Feeding characteristics: Parents were asked to complete the Montreal Children's Hospital Feeding Scale (MCHFS) (33), which was translated and verified using back translation methodology. The MCHFS consists of 14 items covering the following feeding domains, with some overlap: oral motor (items 8 and 11), oral sensory (items 7 and 8) and appetite (items 3 and 4). The following items assessed maternal and family perceptions: concerns regarding feeding (items 1, 2 and 12), mealtime behaviors (items 6 and 8), maternal strategies used during feeding (items 5, 9 and 10) and family reactions to child's feeding (items 13 and 14). Each item is rated on a seven-point Likert scale with anchor points at either end. Seven items are scored from negative to positive, and the other seven are scored from positive to negative. The primary feeder marks each item according to frequency or difficulty level of a particular behavior or level of parental concern. The total feeding problem score is obtained by adding the scores for each item after reversing the scores of the seven negative to positive items. Higher scores represent higher difficulty levels. The total raw scores are converted to T-scores, which are further divided into four categories (no difficulties <61, mild difficulties 61–65, moderated difficulties 66–70 and sever difficulties >70). Parents were instructed to complete the questionnaire based on their recollection of their child's feeding behavior at two years of age, thereby standardizing responses to a defined timepoint.

### 2.9 Statistics

All analyses were conducted using Statistical Package for the Social Sciences software version 29 (SPSS Inc., Chicago, IL, USA). The Kolmogorov–Smirnov test was conducted to test the normality of continuous data. Data is presented as mean ± SD (normal distribution), median (interquartile range, IQR) (skewed distribution) or number (percent) (categorical variable). To compare between the TF and CBF groups, we used independent samples *t*-test or Mann–Whitney *U*-test for variables with normal or skewed distribution, respectively, or Pearson's chi-square tests or Fisher's exact tests for categorical variables.

To compare changes over time in growth index *Z*-scores from birth to 2 years of life between the TF and CBF groups, we conducted linear repeated measures mixed model analyses for the change in growth indices. The models were specified with a within-group factor of time, a between-group factor, and the interaction of group with time. The data are expressed as estimated marginal means and standard errors.

## 3 Results

**Figure 1:**
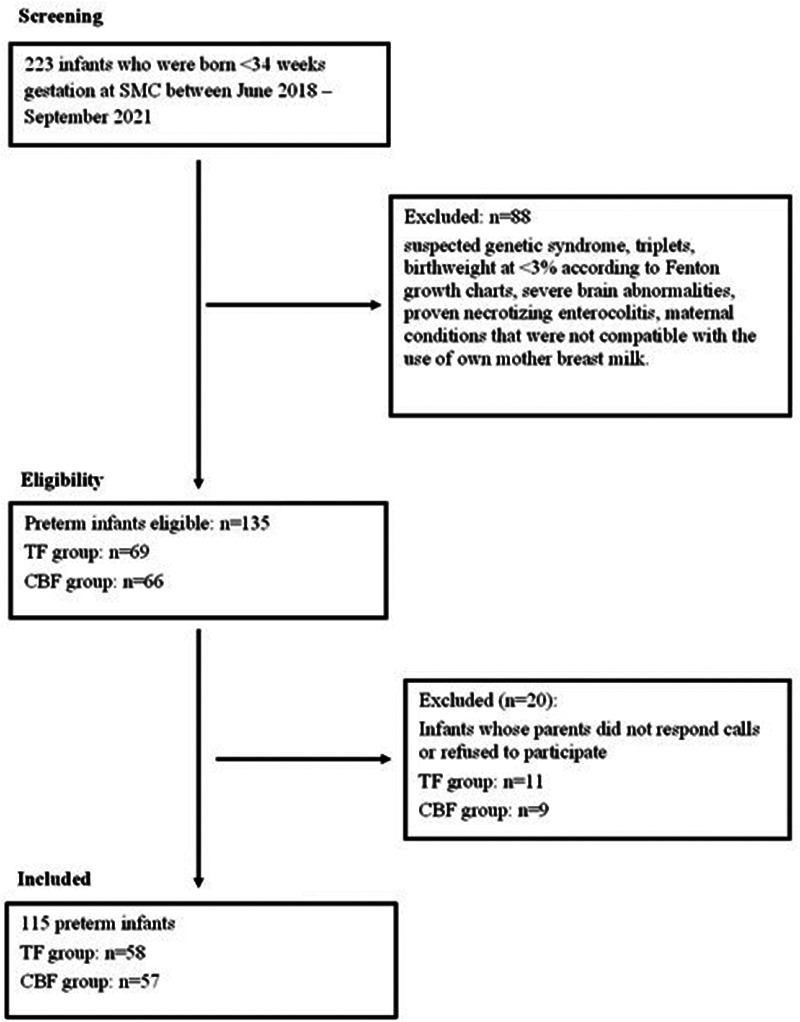
Flowchart of study cohort. TF, traditional feeding, born between June 2018 and February 2020. Intervention group CBF-cue-based feeding, born between January and September 2021.

Of the 223 infants assessed for eligibility, 135 (TF: 69, CBF: 66) met the inclusion criteria. Parents of 115 (85%) infants (TF: 58 and CBF: 57) completed the study questionnaires. Of these, 51parents from each group provided a copy of the child's anthropometric data as measured during visits to the national maternal child health clinics (Figure 1).

Maternal and infant demographic, clinical and anthropometric characteristics during primary hospitalization are shown in Table 1. Maternal demographic and pregnancy characteristics were comparable except for significantly higher rates of surgical delivery among the CBF group compared to the TF group [45(78.9%) vs. 31(53.4%), *p* = 0.004]. The groups were also comparable in terms of gestational age [median (IQR) 31.5 weeks (29.5, 32.2) vs. 31.5 (29.3, 32.8), *p* = 0.440], birth weight [median (IQR) 1,521 gr (1,242, 1,736) vs. 1,583 gr (1,210, 1,740), *p* = 0.506] as well as head circumference and the related *Z*-scores. Apgar scores at 1′ and 5′, need for invasive mechanical ventilation and need for oxygen treatment were also comparable between the groups. The CBF group was discharged at a significantly earlier PMA [median (IQR) 35.5 (35.2, 36.2) vs. 36.9 (36.1, 38.6) *p* < 0.001] and had a significantly shorter length of stay (LOS) (mean of 11.8 days) compared to the TF group. At discharge, the CBF group and the TF group had a similar number of mother's milk portions per day [median (IQR): 4 (0, 7) vs. 3 (0, 6) *p* = 0.919], but the CBF group had a greater number of breast feedings per day [median (IQR): 1 (0, 1) vs. 0 (0, 0) *p* = 0.003]. Additionally, the CBF group had a lower absolute weight and head circumference. Weight and head circumference *Z*-scores at discharge were comparable between the groups.

**Table 1:** Demographic and clinical characteristics of the study population.

	TF (*n* = 58)	CBF (*n* = 57)	*P*
Maternal and pregnancy characteristics			
Age (year) Mean ± SD	30.7 ± 5.4	31.2 ± 5.3	0.578
Married *n* (%)	55 (94.8)	53 (98.1)	0.619
Smoking *n* (%)	10 (17.2)	2 (3.6)	0.019
Chronic illness *n* (%)	14 (24.1)	19 (33.3)	0.276
Previous deliveries Median (IQR)	1 (0, 2)	0 (0, 2)	0.725
Twin pregnancy *n* (%)	24 (42.1)	16 (27.6)	0.102
Surgical delivery *n* (%)	31 (53.4)	45 (78.9)	0.004
Infant characteristics			
Gestational age (weeks) Median (IQR)	31.5 (29.5, 32.2)	31.5 (29.3, 32.8)	0.440
Birth weight (g) Median (IQR)	1,521 (1,242, 1,736)	1,583 (1,210, 1,740)	0.506
Birth weight Z score Mean ± SD	−0.11 ± 0.72	0.004 ± 0.77	0.413
Head circumference (cm) Median (IQR)	28.3 (26.5, 30.0)	28.5 (26.7, 30.5)	0.422
Head circumference *Z* score Mean ± SD	0.12 ± 0.78	0.29 ± 0.79	0.247
Male *n* (%)	37 (63.8)	28 (49.1)	0.113
Apgar 1-min *n* (%)			
7–10	52 (89.7)	48 (84.2)	0.450
4–6	3 (5.2)	7 (12.3)	
<4	3 (5.2)	2 (3.5)	
Apgar 5-min *n* (%)			
7–10	58 (100)	55 (96.5)	0.243
4–6	0	1 (1.8)	
<4	0	1 (1.8)	
Mechanical ventilation *n* (%)	18 (31.0%)	10 (17.5%)	0.093
Oxygen duration (day) Median (IQR)	3 (0, 12)	2 (0, 5.5)	0.391
Age at discharge (weeks) Median (IQR)	36.9 (36.1, 38.6)	35.5 (35.2, 36.2)	<0.001
Hospitalization length (days) Mean ± SD	45.2 ± 19.2	33.4 ± 16.6	<0.001
Weight at discharge/36 weeks (g) Median (IQR)	2,485 (2,223, 2,848)	2,154 (2,015, 2,395)	<0.001
Weight *Z* score at discharge/36 weeks Mean ± SD	−0.92 ± 0.74	−1.00 ± 0.69	0.539
Head circumference at discharge/36 weeks (cm) Median (IQR)	33.0 (32.1, 33.7)	32.0 (31.3, 32.8)	<0.001
Head circumference *Z* score at discharge/36 weeks Mean ± SD	−0.22 ± 0.73	−0.28 ± 0.77	0.702
Number of mother's milk portions per day Median (IQR)	3 (0,6)	4 (0,7)	0.919
Number of breast feedings per day Median (IQR)	0 (0,0)	1 (0,1)	0.003

TF, traditional feeding; CBF, cue based feeding.

Data is presented as mean ± SD (normal distribution), median (interquartile range, IQR) (skewed distribution) or number (percent) (categorial variable).

*P* values represent independent samples *t*-test or Mann–Whitney *U*-test, for variables with normal or skewed distribution, respectively; or Pearson's chi-square tests or Fisher's exact tests for categorial variables.

Table 2 shows the repeated-measures mixed-model analyses for changes in growth indices (weight, length, BMI, head circumference *Z*-scores) during the first 24 months of life. Median corrected age at measurements were comparable between the CBF and the TF groups at 12 months [median (IQR): 10.7 (10.2, 11.6) vs. 10.4 (10.0, 10.9) months, *p* = 0.124], at 18 months [median (IQR): 16.4 (16.2, 17.0) vs. 16.4 (16.0, 17.1) *p* = 0.792], and at 24 months (actual age) [median (IQR): 26.5 (25.3, 27.6) vs. 26.2 (25.2, 28.1) *p* = 0.773]. All mean growth indices for both groups were within the normal range (>−2 SD).

**Table 2:** Changes in growth indices from birth to 24 months in the TF and CBF groups.

	Birth	12M	18M	24M	Ptime	Pgroup	Ptime × group
Weight *Z*-score							
TF	−0.11^a,b^ (0.12)	0.04^b^ (0.14)	0.18^b^ (0.14)	−0.41^a^ (0.15)	0.023	0.004	0.084
CBF	−0.01^a^ (0.14)	−0.37^a,b^ (0.16)	−0.38^a,b^ (0.19)	−0.77^b^ (0.18)	0.010		
Length *Z*-score							
TF		−0.26^a^ (0.16)	−0.23^a^ (0.16)	−0.81^b^ (0.17)	0.027	0.003	0.762
CBF		−0.47^a^ (0.21)	−0.73^a,b^ (0.26)	−1.12^b^ (0.24)	0.071		
BMI *Z*-score							
TF		0.23 (0.15)	0.45 (0.15)	0.08 (0.16)	0.229	0.025	0.683
CBF		−0.10 (0.15)	0.03 (0.19)	−0.06 (0.18)	0.869		
Head circumference *Z*-score							
TF	0.13 (0.11)	0.11 (0.14)	0.35 (0.14)		0.374	0.227	0.086
CBF	0.29 (0.13)	−0.03 (0.15)	−0.10 (0.18)		0.113		

TF, traditional feeding; CBF, cue-based feeding.

Linear repeated measures mixed model for the change in growth indices *Z*-score from birth to 2 years. Data is presented as estimated mean and standard error (SE). Rates with different superscripts (a, b) differ significantly from each other (*P* < 0.05). Interpretation: Although absolute *Z*-scores were lower in the CBF group, no significant time-by-group interaction was observed for any growth parameter, indicating that growth trajectories over time were parallel between the groups.

Weight *Z*-scores: For the TF group, mean weight-for-age *Z*-scores increased slightly from birth (−0.11 ± 0.12) to 18 months (0.18 ± 0.14), and then declined by 24 months (−0.41 ± 0.15) (Ptime = 0.023). The CBF group showed a continuous decline over time, from −0.01 ± 0.14 at birth to −0.77 ± 0.18 at 24 months (Ptime = 0.010). The TF group had higher overtime mean weight *Z*-scores than the CBF group (Pgroup = 0.004); the time-by-group interactions were not statistically significant (Ptime × group = 0.084).

Length *Z*-scores: In both groups, length-for-age 0-scores declined over time. TF infants decreased from −0.26 ± 0.16 at 12 months to −0.81 ± 0.17 at 24 months (Ptime = 0.027), and CBF infants declined from −0.47 ± 0.21 to −1.12 ± 0.24 (Ptime = 0.071). The TF infants had higher overtime mean length *Z*-scores than the CBF infants (Pgroup = 0.003), with no significant time-by-group interaction (slope of change) between the groups (Ptime × group = 0.762).

BMI *Z*-scores: TF infants had significantly higher BMI *Z*-scores than the CBF group (Pgroup = 0.025), with no significant overtime change in either groups (Ptime = 0.229 and Ptime = 0.869 in the TF and CBF groups, respectively) with no time-by-group interaction (Ptime × group = 0.683).

Head Circumference *Z*-scores: Head circumference-for-age *Z*-scores remained stable in both the TF and the CBF groups (Ptime = 0.374 and Ptime = 0.113, respectively). No statistically significant differences were observed between groups (Pgroup = 0.227), with no time-by-group interaction (Ptime × group = 0.086).

Table 3 shows individual item scores as well as the mean total score and the difficulty categories of MCHFS of both groups. The TF group scored significantly higher in “force to eat” (item 10) (2.50 ± 2.07 vs. 1.68 ± 1.55, *p* = 0.018) and “influence relation” (item 13) (2.09 ± 1.63 vs. 1.51 ± 1.42, *p* = 0.044) and a trend toward “long mealtimes” (item 5) (2.53 ± 1.10 vs. 2.12 ± 1.20 *p* = 0.057) and “poor chewing” (item 11) (2.55 ± 1.76 vs. 1.98 ± 1.71, *P* = 0.081). The groups were comparable in terms of total score as well as the rate of any feeding difficulty (29.3% and 17.5% of the TF vs. CBF respectively, *P* = 0.137).

**Table 3:** Comparison of scores on the montreal children's hospital feeding scale between the TF and CBF groups.

	TF (*n* = 58)	CBF (*n* = 57)	*P*
1. Difficult mealtimes*	3.21 ± 1.99	3.02 ± 2.13	0.623
2. Worries about feeding	3.66 ± 2.41	3.60 ± 2.45	0.897
3. Poor appetite*	2.26 ± 1.64	2.39 ± 1.77	0.690
4. Start refusing food*	2.79 ± 1.81	2.60 ± 1.90	0.571
5. Long mealtimes	2.53 ± 1.10	2.12 ± 1.20	0.057
6. Bad behavior	2.19 ± 1.66	2.04 ± 1.67	0.619
7. Gags/spits/vomits	2.71 ± 2.05	2.46 ± 2.12	0.521
8. Holding food in mouth*	1.84 ± 1.50	1.58 ± 1.44	0.334
9. Follow around/distract	2.93 ± 2.35	2.86 ± 2.36	0.871
10. Force to eat*	2.50 ± 2.07	1.68 ± 1.55	0.018
11. Poor chewing	2.55 ± 1.76	1.98 ± 1.71	0.081
12. Poor growth*	2.41 ± 1.79	2.37 ± 2.09	0.901
13. Influence relation*	2.09 ± 1.63	1.51 ± 1.42	0.044
14. Influence family relations	1.88 ± 1.56	1.47 ± 1.42	0.146
Total score	35.6 ± 14.7	31.7 ± 16.5	0.184
Total T-score	52.3 ± 11.5	49.4 ± 12.9	0.200
Any feeding difficulty	17 (29.3%)	10 (17.5%)	0.137
No difficulties *n* (%)	41 (70.7%)	47 (82.5%)	0.489
Mild difficulties	8 (13.8%)	4 (7.0%)	
Moderate difficulties	4 (6.9%	2 (3.5%)	
Severe difficulties	5 (8.6%)	4 (7.0%)	

TF, traditional feeding; CBF, cue based feeding.

Data is presented as mean ± SD. *P* values represent independent samples *t*-test for the comparison of individual item score and total score or Pearson's chi-square tests or Fisher's exact tests for the comparison of difficulties categories.

Clinical relevance: Differences observed in coercive feeding behaviors and relational tension domains support CBF as a relationally protective feeding approach.

*Reversed item.* *P value represents Fisher Exact test.

## 4 Discussion

This study extends the current understanding of cue-based feeding (CBF) by demonstrating its potential benefits beyond the neonatal hospitalization period. In this cohort of preterm infants, CBF was associated with earlier attainment of discharge readiness and a shorter length of stay, while supporting normal growth patterns across the first two years of life. Importantly, CBF was also linked with fewer coercive feeding behaviors and lower relational tension during mealtimes, suggesting that its impact is not solely nutritional but also relational and developmental.

The reduction in length of hospital stay is consistent with previous research showing that responsive, infant-led feeding supports more efficient acquisition of oral feeding skills (15–18, 34). Earlier studies reported inconsistent effects on hospitalization duration (18), likely due to variation in implementation strategies and parental involvement. Our findings add to growing body of evidence that when CBF is implemented through a structured, parent-partnered model, it can shorten hospitalization without compromising discharge anthropometric indices. Given the well-established association between prolonged hospitalization, parental stress, reduced confidence, and impaired bonding (35, 36), such reductions have meaningful implications for both families and NICU systems of care.

Growth trajectories among preterm infants are known to differ from those of term-born peers, with catch-up growth typically occurring between 24 and 59 months of corrected age (37–39). In our cohort, both groups maintained growth within the normative range throughout follow-up. The somewhat lower weight- and length-for-age *Z*-scores in the CBF group may reflect post-discharge parental adherence to satiety cues or variability in feeding practices, which were not assessed. Notably, the time-by-group interaction was not significant, emphasizing that overall growth patterns were preserved. These findings align with previous longitudinal analyses demonstrating variability in growth among preterm infants that does not necessarily reflect adverse nutritional status (40, 41). Continued structured nutritional monitoring is therefore recommended when implementing responsive feeding models.

Feeding difficulties occur in up to 40% of preterm infants and are associated with malnutrition, growth faltering, and developmental and communication challenges (19, 20, 41–44). Historically, such difficulties were attributed primarily to oral immaturity or procedural trauma. More recent research emphasizes the role of caregiver emotional state, feeding cognitions, and relational patterns in shaping feeding outcomes (10, 45–47). In NICU environments, staff-led, volume-driven feeding may inadvertently undermine parental confidence and responsiveness (19, 43). Our findings showing higher coercive feeding behaviors and greater relational tension in the TF group support the conceptualization of CBF as a developmentally supportive caregiving model. By encouraging parents to observe, interpret, and respond to infant cues, CBF may mitigate maladaptive intrusions and promote co-regulation during feeding. These findings align with the broader responsive feeding framework, in which the goal of feeding is to support the infant's capacity to regulate appetite, behavioral state, and interaction with the caregiver. Rather than prioritizing completion of preset volumes, the caregiver is guided to observe the infant's readiness and satiety cues and respond in a manner that maintains physiological stability. Importantly, this model does not preclude close nutritional monitoring. In our unit, parents and clinicians shared responsibility for ensuring adequate intake, enabling flexibility while maintaining metabolic safety. This balance between nutritional adequacy and responsive interaction may help explain the preserved growth trajectories and reduced coercive feeding behaviors observed in the CBF group.

This interpretation aligns with the sensitive window framework: preterm infants experience a critical phase of neurodevelopment ex utero, during which early parent-infant interactions shape emerging regulatory capacities (48). Interventions that enhance attunement and shared regulation during feeding may therefore confer long-lasting relational and developmental benefits. CBF may be understood as an approach that simultaneously supports physiologic stability, parental confidence, and adaptive feeding relationships.

### 4.1 Limitations

This study has several limitations. Its retrospective, non-randomized design introduces potential confounding, and recall bias is possible because infants in the TF group were older at follow-up. Although a prospective randomized non-inferiority trial would provide a higher level of evidence and allow for stronger causal inference, the transition to cue-based feeding in our unit was implemented as a practice-wide change, precluding randomization. The retrospective design may also limit generalizability and external validity. Post-discharge feeding practices and socioeconomic characteristics were not assessed. The single-center design may limit generalizability, although it enhances internal consistency of care. No standardized readiness assessment tool was used, which may limit reproducibility; however, this decision aligns with responsive care models that prioritize parental attunement over scoring systems (27).

### 4.2 Conclusion

Cue-based feeding was associated with shorter hospitalization and more adaptive parent–infant feeding interactions, while supporting normal growth through two years of age. Integrating responsive caregiving with structured nutritional monitoring may optimize both somatic and relational outcomes in preterm infants. These findings support broader implementation of CBF as part of developmentally supportive, family-integrated NICU care.

## Data Availability

The raw data supporting the conclusions of this article will be made available by the authors, without undue reservation.
